# “You look like them”: Drawing on Counselling Theory and Practice to Reflexively Negotiate Cultural Difference in Research Relationships

**DOI:** 10.1007/s10447-016-9277-4

**Published:** 2016-10-13

**Authors:** Lorena Georgiadou

**Affiliations:** Counselling, Psychotherapy and Applied Social Sciences, School of Health in Social Science, University of Edinburgh, Edinburgh, UK

**Keywords:** Qualitative interviews, Intercultural research, Intercultural counselling, Reflexivity, Power

## Abstract

Located within a context of intercultural counselling research, this paper highlights the pertinence of the researcher’s reflexivity and cultural awareness in relation to research relationships. It draws on an excerpt between a white European interviewer and an Asian trainee counsellor discussing the latter’s experience of intercultural counselling practice. A reflexive analysis of a short passage aims to demonstrate how explicit negotiation of cultural difference within the interview setting advanced the researcher’s understanding of the participant’s experience, which was being investigated. The interrelated challenges, but also the importance of the interviewer’s preparedness to explore power imbalances in the research relationship are also examined, as are some key limitations of such endeavours. This paper underlines the usefulness of counselling skills in qualitative research, hoping to function as an invitation for more practitioner involvement in therapeutic inquiry.

## Linking Counselling Research and Practice

The gap between counselling research and practice is well documented and long-standing (Bondi and Fewell 2016; Finlay 2011; McLeod 2014), despite recent attempts by professional bodies to strengthen the field’s ‘research base’ by making research a critical component of professional qualifications and accreditation (Letourneau 2015, p. 375). Scholars suggest that practitioners’ lack of interest in research is linked to a domination by positivist paradigms in research that have little relevance or applicability to therapeutic practices and processes (Bondi and Fewell 2016; Finlay 2011; Letourneau 2015). As a response, the field is slowly moving towards a recognition of epistemologies and methodologies that are more in line with counselling values and practice, such as qualitative (e.g., Etherington 2004; Finlay 2011) and ‘experience-near’ (e.g., Bondi and Fewell 2016) approaches to inquiry that acknowledge practitioner wisdom (*ibid*) and “generate practical knowledge that makes a difference” (McLeod 2014, p. 10).

Counselling research, like practice, is often phenomenological, as it is generally concerned with people’s subjective experiences and with how people understand and make meaning of various aspects of their lives and their relationships (Etherington 2004; Finlay 2011; Josselson 2013). One common way of gaining knowledge about individuals and their lives is by engaging them in qualitative interviews, where their stories can be explored in depth (Roulston 2010). In therapeutic inquiry, qualitative interviews typically provide insight into relevant individuals’ (e.g., clients, counsellors, trainee counsellors, supervisors) experiences and practices, enabling their ‘voices’ to be heard, and, consequently, to have an impact on counselling training, practice and policies (McLeod 2014).

Counselling and qualitative interviews have many things in common and, although they are distinct practices, parallels can be drawn between the two. Both therapeutic practice and qualitative interviews are ‘projects of making meaning’ (Bondi 2013, p. 9) that involve two people who meet and engage in some sort of dialogic interaction, with one interlocutor in a (mainly) listening role, seeking to understand more about the other person (Finlay 2011). Some scholars even draw attention to the potentially therapeutic nature of research interviews for participants who may find the invitation to tell their story in the presence of an interested other, beneficial (Birch and Miller 2000; Bondi 2013; Josselson 2013). They also highlight the interrelated risks of these similarities, though, such as the possible blurring of boundaries between the two situations, resulting in participants who overshare and “get too personal” (Josselson 2013, p. 36) and therapists-interviewers who find it hard to stay focused on the research and refrain from making therapeutic interpretations or wanting to help in some way (Finlay 2011; Josselson 2013).

Despite the potential challenges, the similarities of the two practices create opportunities for therapists in research roles. As Finlay (2011, p. 7) suggests, therapists have “distinct advantages over other professionals”, as they bring “valuable professional competencies” to research interviews. Some of these competencies relate to skills that counsellors develop within training, such as listening attentively and with curiosity, summarising and probing for deeper explorations, being empathic and offering an accepting presence that creates a safe environment for people to share their stories (Finlay 2011; McLeod 2014). In addition to these practical skills, counsellors are also considered ‘experts’ in reflexive practice (Etherington 2004, 2007). This can be immensely advantageous in social science (qualitative) research, where the reflexive use of self has increasingly become more ‘legitimate’ (Etherington 2007), to the point where it is even considered a “defining feature of qualitative research” (Finlay 2003, p. 5).

While reflexivity is widely recognised as an essential component of qualitative research, reflexive analyses of relational aspects of the research process itself rarely find their way to publication. Theoretically, researchers are encouraged to reflect on research relationships (Bondi 2003) and “display in our writings/conversations the interactions between our selves and our participants from the first point of contact until we end those relationships” (Etherington 2007, p. 601). Yet the focus of published scholarship seems to be on disseminating *findings,* rather than exploring how these findings are generated. Put differently, it is seldom clear how researchers ‘do’ reflexivity in relation to self and other (Gough 2003). This paper suggests that counsellors have a lot to offer in that respect, with the subsequent section discussing how aspects of counselling practice can advance research practice.

## Reflexivity in Counselling and Research Practice

In social science research, the researcher’s reflexivity is loosely understood as meta-analysis or self-awareness; that is, the ability to think about one’s own stance, identity, bias and context, and consider how these may influence the research process and outcome (Etherington 2007; Gough 2003; Willis and Stiltanen 2009). In therapeutic work, however, reflexivity goes well beyond self-awareness. Reflexive practitioners also notice, monitor and, if appropriate, openly explore the therapeutic relationship to facilitate the therapeutic process and/or advance their understanding of their client’s world (Etherington 2004, 2007; McLeod 2014; Reupert 2006).

In relational therapies particularly, practitioners learn a great deal about their clients and how they may relate to others by paying close attention to how clients relate to them, what happens in the room, what is being said or left unsaid, and by noticing their own responses to clients. It is suggested here that such reflections, explorations and negotiations of the ‘here-and-now’ relationship and the dynamics of the interaction in the research context, could greatly advance research practice and the researcher’s understanding of the participant’s world (Josselson 2013). Put simply, qualitative researchers have a lot to gain from moving beyond self-awareness to more targeted explorations of their evolving research relationships. And, as argued, the wider field of social science research can also benefit from such considerations and publications.

On one level, researchers could learn a lot about their participants and “the contexts in which they construct their lives” (Josselson 2013, p. 37) by noticing how they and their participants perceive, respond and relate to each other in the interview setting – the “interview dance” as Josselson (2013, p. 30) beautifully described it. This becomes particularly pertinent in studies where the focus of investigation is on relational processes, as it often is in counselling research. Finlay and Evans (2009) conceptualised such practices as relational-centred research and pointed to the usefulness of endorsing values and stances common in therapeutic practice, such as having an open presence, attending to embodied intersubjectivity, acknowledging dialogic co-creation of meaning as well as the existence of multiple and entangled selves.

Peeling one more layer off, researchers can also benefit greatly by another aspect of therapeutic practice; that is, by being aware of and sensitive to the power dynamics that take place in interviews (Finlay 2003). Just like in counselling, the interviewer’s ability to notice, handle and negotiate power imbalances and to remain sensitive to cultural difference in the research environment are key to good research practice (Cloke et al. 2000). Increasingly, researchers move away from a naïve, rather utopian (Ben-Ari and Enosh 2013) belief that power imbalances can be eradicated through egalitarian practices (e.g., by interviewing participants with whom they share identities/characteristics; by involving participants in the analytic process). Today most post-modernist researchers accept that it is ‘virtually impossible’ to avoid inequalities in the research relationship. On the one hand, researchers are often seen as ‘intellectual authorities’ who design and control the project, the environment and the direction of the dialogue (Gadd 2004; Gough 2003). On the other, participants are not entirely powerless either, as they have the right to consent to or withdraw participation and have control over the material they share (Kvale 2006). A more pragmatic stance is, therefore, endorsed, whereby egalitarian practices are welcomed, but asymmetries are acknowledged, reflected upon and considered as a source of knowledge (Ben-Ari and Enosh 2013; Kvale 2006).

As discussed earlier, there is little guidance available on how to ‘do’ reflexivity and how to handle constructively power asymmetries in research settings, and it is suggested here that therapists have a lot to offer in this direction. Counsellors are trained to recognise difference and diversity and to acknowledge and negotiate power issues between themselves and their clients, rather than being oblivious to them (e.g., Proctor 2002). Skillful negotiation of power issues in the therapy room can not only facilitate the client’s process and in-therapy experience, but can also advance the counsellor’s understanding of her client’s experience in society in relation to difference and diversity (Dupont-Joshua 1996). Similarly, the researcher’s skillful handling of power asymmetries does not only contribute to ethical research practice, but can also offer insight into the participant’s experience of diversity and power in other contexts. For example, in a similar way that a counsellor may explore her client’s response to aspects of her own identity (e.g., gender or religion) to explore the influence of power imbalances on the therapeutic process or her client’s wider views on or experiences of difference and diversity in society, an interviewer could pay attention to, or even explicitly discuss, diversity issues that are present in the research environment. This could be done to explore the impact of power dynamics on the research process and outcome, or to investigate the participant’s views on or experiences of difference in another, relevant to the inquiry, context.

The above juxtaposition of counselling and research reveals a number of advantages associated with the involvement of counsellors in research interviews, both for the domain of counselling and for the wider field of social science research. On the one hand, acknowledgment of the “distinctive advantages” (Finlay 2011) that therapists have in the researcher role may encourage potentially hesitant practitioners to engage in research. Similarly, wider recognition of the usefulness of practitioner wisdom and attention to the relational aspects of research practice may also inspire more counsellors to engage with research material. This is likely to reduce the research-practice gap and advance the overall profession. Importantly, exploration and negotiation of the research relationship and the interrelated power dynamics can be particularly pertinent to projects that investigate relationships and interactions, a common phenomenon in therapeutic inquiry.

On the other hand, consistent and widespread engagement of counsellors in reflexive, relational-centred research practice is likely to result in the production of publications that value reflexivity as a process and that disseminate examples of ‘doing’ reflexivity, which is rare in existing literature. So ultimately, counselling research may pave the way for robust, case-based, process-sensitive and relational-centered scholarship that can function as a model for other disciplines in the social sciences.

To illustrate ‘doing reflexivity’, and the use of the research relationship to better understand the participant’s experience of what was being investigated, a case example is provided. The example involves research where the research relationship and, in particular, a specific manifestation of cultural difference between interviewer and interviewee and its negotiation during the interview, offered a great opportunity for furthering the researcher’s understanding of her participant’s experience in relation to the phenomenon that was being investigated. It involves a case of reflexive processing of this negotiation, ultimately highlighting the usefulness of counselling skills in interview-based research.

## Personal Location

Perhaps opposite to what might be expected (given the preceding argumentation), this is not an example where a counsellor used professional experience to skillfully explore and negotiate a research relationship. Instead, it is one involving a researcher (the author) being unprepared for acknowledging power differences that were present in the interview setting and her clumsy attempt to negotiate the power imbalance. The central thesis presented within this article crystallised from a reflexive exploration of my role as a researcher within the domain of counselling, without being a trained or practising counsellor, and my subsequent conclusion of the potential benefits of therapeutic skills for research practice.

Prior to presenting the context of the research from which this vignette was extracted, it is important to acknowledge the somewhat narrow focus of the ‘incident’ depicted, focusing as it does on difference around the ‘visibility of ethnicity’. Such a narrow focus comes with limitations and perhaps even reinforces stereotypical understandings of ‘multiculturalism’ and power imbalances as being only associated with race and ethnicity. The reason for selecting this example is merely the simplicity and powerfulness of the particular incident, which resonated with the author for a long time.

As an element of the title of this present article suggests (“You look like them”), a research participant ‘othered’ me, as the interviewer, in an explicit way by commenting on the visibility of our ethnic difference. I believe I handled this unexpected confrontation rather awkwardly due to my naiveté around the impact of my cultural identity (being a Greek national, but involving my whiteness in particular) on the research. This made me reflect on the usefulness of counselling skills like cultural awareness, the ability to use the ‘here and now’ relationship to gain insight into the other person’s experience, and to acknowledge and negotiate difference in the interview setting in a robust manner. I hope that dissemination of this case will function as an example of how one might ‘do’ reflexivity in qualitative research, while highlighting the potential usefulness of therapeutic skills and values for qualitative research more broadly.

## The Context of the Research Study

The doctoral study from which this work and the example derives was born out of the desire to investigate and understand international trainees’ experiences of counselling practice in unfamiliar cultural and/or linguistic contexts (Georgiadou 2014, 2015). From this, ‘cultural difference’ was examined through a subjective prism of what has been described as a sense of ‘alterity’ (Smith 2015) or of ‘not being from here’ (Kissil et al. 2013).

Two groups of international counselling trainees (native and non-native English speakers) were interviewed about their experiences of beginning counselling practice in a second language and/or an unfamiliar culture. The semi-structured interviews explored participants’ thoughts, feelings and experiences of working with clients in a language other than their mother tongue (for non-native speakers) and/or in an unfamiliar culture, where the use of English may be very different to what they were used to (for native speakers). The interviews were then analysed following the principles of Smith et al.’s (2009) Interpretative Phenomenological Analysis. As the sample recruitment was based on linguistic criteria, language use was at the core of the investigation. Nonetheless, participants were invited to discuss any aspect of their perceived ‘alterity’ - the experience of ‘not being from here’ - they wished to.

Two of the non-native speaking participants were Asian, and for one of them, Claire (pseudonym), the visibility of her ethnicity was a preoccupation which, as to be explained, was articulated and elaborated through a direct comparison between herself and me, as the interviewer.

## Presentation of the Case

Claire was an Asian participant who came to the UK to train as a counsellor. At the time of the interview she had been in the UK for eighteen months and had been practising as a counsellor in an agency for approximately eight months. When asked about her experience of seeing clients in the UK, Claire mentioned that she was a visible *‘ethnic minority’* and that she worried that she might get stereotyped as being *‘unprofessional’* and *‘naïve’*, attributes that she mentioned as being the *‘typical stereotypes’* for Asian people in Western societies. The following extract constitutes an illustrative example of Claire’s position in relation to the visibility of her ethnicity in practice:

I fear that the client might think that “how do you understand me, you are from far away” like say that “you are not Caucasian...”

This excerpt is relatively self-explanatory: Claire seems concerned that her clients may doubt her professional competence and reject her due to the visibility of her ethnicity (“*you are not Caucasian”*). Given the commonality of “internalised racism” or “internalised oppression” in the counselling literature (Alleyne 2004) and the project’s interest in *experiences* of intercultural counselling (with a focus on language use), I, as the interviewer, was eager to move beyond Claire’s ‘fears’. Admittedly, Claire’s experience (which may have not included any specific incident of ethnic discrimination, but which was very much shaped by her own concerns around it) was at risk of being ignored. Importantly, however, Claire insisted:

...it’s like even though... like you are from Greece? [I: mhmm] but you are still in... Euro-zone, but for me if I- I’m totally different, like from far away, non-white, kind of... minority background.

While talking about her experience in practice, she suddenly brought my nationality into the discussion and made a direct comparison between us *(“you are still in Euro-zone”, “I’m totally different”*). While still subtle, this indicated that Claire did not see me in terms of sameness. While we were both non-British, non-native English speakers, our foreign identity incorporated a differentiating factor: the visibility of our ethnicity. I was European and white, while she was “*from far away”*, *“non-white”* and from a “*minority background”*. This differentiating position clearly revealed Claire’s self-perception in the predominantly white, European environment in which she was practising.

In my view, this direct comparison facilitated Claire’s disclosure of her experience of feeling different more directly, including within the counselling domain that was being investigated. It presented her with a concrete example in which to frame her perhaps difficult to express experience of alterity. This supports the suggestion that paying attention to the ‘here and now’ research relationship and negotiating power imbalances in the research setting can offer insight into participants’ experiences in different contexts – just like negotiation of the therapeutic relationship can illuminate clients’ patterns of behaviours and ways of relating to others.

Claire’s direct comparison kindled my interest in reflecting upon power dynamics in the interview setting. This has subsequently enhanced my reflexive stance and advanced, not only my data analysis work, but also my self-awareness. The acknowledgement of difference and the role attribution of ‘white majority’ came as a surprise to me, as visibility of ethnicity was not the cultural aspect that I would have identified as salient in my encounter with Claire. Given the centrality of second language use in the project, the comparative feature that I, as the researcher, was focusing on was language. Since we were both non-native English speakers in a predominantly English-speaking environment, I experienced us as being ‘equal’ and expected us to face similar difficulties in cross-cultural encounters – difficulties that would be associated with second-language use.

Intrigued by Claire’s acknowledgement of difference, I asked her to elaborate:

I: so you think that someone like me, for example, who I come from Europe and I still have English as a second language would-

C: I think that it is easier for you to work...

I: to work with clients? [C:mhmm, mhmm...] Why?

Scrutiny of my choice of words here reveals my own agenda to focus on language (*“and I still have English as a second language”*). It also shows my attempt to deflect the focus of this dialogue from ‘me’ to *‘someone like me’*, a European, white, non-native English-speaking individual. On one level this was the outcome of feeling uncomfortable ‘taking on’ the counselling practitioner role that I felt (or, arguably I feared) my participant was potentially attributing to me (*‘it is easier for you to work [with clients]’),* as I was not a trained counsellor. Upon reflection, however, I realised that I was also attempting to get some distance from the personal turn that this dialogue had started taking, as I was uncomfortable with the attribution of the ‘white majority’ role, inherent in Claire’s words.

At this point a parallel between theoretical views on intercultural counselling and this particular interview experience can be helpful. Ryde (2011) has suggested that white practitioners often refuse to take on attributed roles of ‘whiteness’ out of guilt and shame; she also pointed out that the therapist’s whiteness is usually so common that there is often nothing about it to discuss. Similarly, my attitude here (of focusing centrally on language) suggested that I, too, may have been ashamed of my whiteness, or taken it for granted in that context. My subsequent surprised reaction (“…*to work with clients?*”) and my reluctance to understand or perhaps accept what Claire was telling me (my follow-on query *“Why?”*) illustrates what has been described in the relevant literature as ‘race avoidance’; i.e., a naively adopted position of not seeing people in terms of race and ethnicity but, rather, as human beings (Thompson and Jenal 1994). Indeed, to my mind, if both Claire and I were non-native English speakers, I would not expect to face fewer difficulties in counselling practice just because I was white.

In retrospect, I realise that this way of thinking revealed my failure to acknowledge my ‘whiteness’, in comparison, and the evident difference in the research relationship. Perhaps an experienced counsellor in the interviewer’s role, with presumably heightened self- and cultural-awareness would have been able to recognise and accept the significance of ethnic difference for Claire, and would have been able to negotiate this in a more skillful manner. Instead, my naïveté invited Claire to respond so simply, yet so powerfully, to make herself understood:

C: Because you look like them!

In terms of power dynamics in the interviewing relationship, this statement distanced me from her directly and positioned me on ‘the other side’ – among “*them”*. This shook my understanding of our research relationship, which, to my mind was essentially equal. As explained, I perceived myself as being one of ‘us’ (non-British, non-native speakers) and not one of “them” (“white majority”). No matter what I thought, however, about our ‘equal’ positions, Claire made explicit that for her we were different, because I “*look like them”*. Even if I was not willing to accept it and was trying to find a way around it, our visible difference was there, occupying space in the room.

Linking back to the previously discussed positions about power asymmetries in research relationships, Claire helped me realise the impossibility of ‘equalising’ power in an interview setting. Moving away from an egalitarian attitude and developing a more accepting position in relation to power and difference, I came to understand that a researcher can perhaps approach participants in a respectful way and attempt to balance out some of the unequal parameters, but can never ensure a perfectly egalitarian interaction. Power imbalances are inherent in the researcher/participant roles, with an infinite number of identities or traits becoming intertwined and complicating further these interactions. It is by being aware of these differences, by reflecting upon their various roles and identities and by negotiating power asymmetries in the interviewing relationship, that a researcher can strive to appropriately handle those issues and enhance research quality (Gadd 2004; Holstein and Gubrium 2004; Roulston 2010).

As explained, reflexivity and self-awareness are thought to advance interpretations and understanding of the generated data and, therefore, enhance a hermeneutic-phenomenological project’s overall quality. In this case, had Claire not made the explicit comparison that advanced my awareness of my whiteness and made me reconsider my positioning in relation to her, I might have overlooked the significance of the visibility of her ethnicity in her experience of living and practising in Britain. By ‘confronting’ me, Claire found a way to ensure that what was central in her experience of counselling practice (the visibility of her ethnicity) would get acknowledged in a project that was exploring the phenomenon of intercultural counselling, yet with a particular emphasis on language. In short, Claire managed to make the researcher realise that her own agenda was putting an essential aspect of her participant experience at risk of being overlooked.

To summarise, this brief extract from Claire’s interview exposed multiple points for discussion. Firstly, it revealed the centrality of the visibility of ethnicity in Claire’s experience of intercultural counselling practice, promoting the researcher’s understanding of the phenomenon under investigation. Lago and Thomson (1996) suggest that it may not always be easy for research participants to pinpoint and discuss issues of cultural difference in intercultural counselling, be it involving clients, trainees, experienced practitioners, trainers, or supervisors. So, making use of the research relationship may offer hints or elucidate participants’ interrelated experiences in other settings. Secondly, this interaction with Claire illuminated some of the power dynamics that may be present in qualitative interviews, and the complexity of identifying and addressing them adequately, especially when they fall outside the interview’s focus and the researcher’s primary interests.

At the same time, this passage indicated the potential unpreparedness or reluctance of the ‘white’ researcher to accept and recognise the impact of her ethnicity in the research process, as well as the significance of not doing so. Potentially, a counsellor with expertise in paying attention to their interlocutor but also to their own self, practice and the relationship, might hopefully have noticed and been more accepting of cultural differences, identities and their interrelated power imbalances.

## Limitations

The suggestion here to make use of the research relationship to better understand participants’ experiences in other contexts, such as experiences of counselling practice, entails a number of challenges that should be discussed. Firstly, in spite of the context-related similarities between research interviewing and counselling pointed out earlier, there are differences that must not be minimised. Elements of intercultural interaction in the interview setting may not necessarily reflect intercultural counselling processes, but might simply relate to power asymmetries in the research dyad. For example, Claire’s direct opposition to me may be an expression of her power ‘counter control’ as a participant (Kvale 2006), mirroring her nervousness, her reluctance to answer my question, her attempt to contest my authority as an interviewer, and such like.

Secondly, and perhaps more importantly, using the research relationship to explore issues of cultural differences can entail particular risks for participants (and arguably for the researcher too). Depending on individuals’ experiences, cultural difference and intercultural experience can be emotive topics. Claire’s ability to explicitly articulate our ethnic difference in this intercultural interviewing setting was somewhat surprising to me as the interviewer. Not all interviewees would feel comfortable enough to address the interviewer in the direct way that Claire did, given the frequently attributed power to the (white) researcher/interviewer role (Kvale 2006). Additionally, in the same way that not all ‘culturally different’ clients would want to explore diversity issues in therapy, as Lago (2010) suggests, it is possible that not all ‘culturally different’ interviewees would want to address these issues in the interview context. Therefore, bringing cultural difference to the ‘front line’ and addressing differences in the interviewing encounter, can be very appropriate for certain projects, but also raises multiple ethical considerations that require reflection and careful handling.

Finally, one limitation of this work that also calls for attention, lies in its exclusive focus on one aspect of cultural difference, namely visibility of ethnicity, which is admittedly restricting. Culture and, therefore, cultural difference are inevitably multifaceted, leading to the view that intercultural counselling should not be seen as a particular approach but as a ‘philosophy of practice’ embedded in all approaches (Moodley and Lubin 2008). In this regard, it is also acknowledged that exploring multiple socio-cultural attributes and their intersections is important in cultural difference, instead of focusing only on race and ethnicity (see, for example, Moodley and Lubin’s, 2008, discussion on the ‘Big 7’ socio-cultural identities). As Lago (2010, p. 76) suggested, however, focusing on “generalized diversity” can “overshadow the specific complexities raised” in particular settings. This work aimed to highlight one of those ‘specific complexities’, and for this reason, difference was only examined through the prism of the visibility of ethnicity.

## Conclusions

This paper has drawn empirically on an extract from an intercultural interview, where the interviewee used the difference present in the interview relationship to better explain her experience of intercultural counselling practice. This has then offered opportunity for a reflexive discussion of the researcher’s interrelated thoughts, reactions and actions. Overall, it has been argued that in qualitative research, and particularly in phenomenological explorations where the focus is on participants’ subjective experiences and understandings of aspects of their life and relationships, researchers can learn a great deal about their participants by paying attention to the interactions in the interview environment, the way interviewee and interviewer engage and respond to each other, the manifestations of power asymmetries between them, and even the reactions they have to their participants – just like counsellors do with their clients.

Similarly to a therapeutic context, what happens between participant and researcher in the interview setting can be a great source of information about the phenomenon that is being investigated; and the researcher’s reflexivity and ability to explore, and perhaps even explicitly negotiate the research relationship, can improve the quality of the study. As showcased in this article, however, negotiating power imbalances, acknowledging difference and handling inequalities in research contexts does not come without complications. Counsellor skills and experience can be extremely useful in research practice, and wider involvement of counselling practitioners with research could greatly contribute to the development of a robust, experience-near, context-sensitive body of counselling research that is relevant to therapeutic practice, hopefully bridging the relevant gap. At the same time, such developments could also feed into the wider social science research debate, contributing to methodological dialogues on how to ‘do’ reflexivity and offering insights into the research process, in addition to the quality of research outcomes.
